# Extraction and purification of C-phycocyanin from *Spirulina platensis* (CCC540)

**DOI:** 10.1007/s40502-014-0094-7

**Published:** 2014-07-16

**Authors:** Devendra Kumar, Dolly Wattal Dhar, Sunil Pabbi, Neeraj Kumar, Suresh Walia

**Affiliations:** 1 Centre for Conservation and Utilisation of Blue Green Algae, Indian Agricultural Research Institute, New Delhi, 110012 India; 2 Microbiology Department, Kurukshetra University, Kurukshetra, India; 3 Division of Agricultural Chemical, Indian Agricultural Research Institute, New Delhi, India

**Keywords:** *Spirulina*, DEAE-Cellulose-11, Phycocyanin

## Abstract

In this study a simple protocol was developed for purifying phycocyanin (PC) from *Spirulina platensis* (CCC540) by using ammonium sulphate precipitation, followed by a single step chromatography by using DEAE-Cellulose-11 and acetate buffer. Precipitation with 65 % ammonium sulphate resulted in 80 % recovery of phycocyanin with purity of 1.5 (A_620_/A_280_). Thro1ugh chromatography an 80 % recovery of phycocyanin with a purity of 4.5 (A_620_/A_280_) was achieved. In SDS_PAGE analysis, the purified PC showed the presence of two subunit α (16 kD) and β (17 kD).

**Electronic supplementary material:**

The online version of this article (doi:10.1007/s40502-014-0094-7) contains supplementary material, which is available to authorized users.

## Introduction

Cyanobacteria, also known as blue green algae (BGA) are a class of gram negative bacteria, which are considered to be the oldest form of life on the earth. They possess a wide range of coloured components, including carotenoids, chlorophyll and phycobiliproteins. Phycobiliproteins (PBPs) are large water soluble supramolecular protein aggregates involved in light harvesting in these organisms and may comprise as much as 40–60 % of the total soluble protein in these cells (Bogorad 1975). These can be divided broadly into three classes based on their spectral properties: Phycoeryhrin (λ_max_-565 nm), phycocyanin (λ_max_-620 nm) and allophycocyanin (λ_max_-650 nm) (Glazer and Bryant 1975). These are composed of two different kinds of polypeptide of which one is low molecular weight α unit (MW: 12–19 kD) and other is large β unit (MW: 14–21 kD), and are generally present in equimolar amounts (Bernard et al. 1992).

The PBPs, mainly phycocyanin have been widely used as nutritional ingredients, natural dyes, florescent markers (Glazer and Stryer 1984), pharmaceuticals such as antioxidants (Romay and Gonzalez 2000) and anti-inflammatory reagents (Qureshi et al. 1996). Phycocyanin is used as colorant in food (chewing gums, dairy products, gellies etc.) and cosmetics such as lipstick and eye liners in Japan, Thailand and China. It has also shown to have therapeutic value (immuno-modulating activity and anticancer activity) (Lijima and Shimamatsu 1982). Phycocyanin is the most important natural blue pigment used in the food and biotechnology because of their colour, fluorescence and antioxidant properties. Cyanobacteria, as a source of PC are being exploited for a long time. But most studies have focussed on production and purification of PC from *Spirulina platensis* (Boussiba and Richmond 1979; Hirata et al. 2000; Minkova et al. 2003; Yan et al. 2011).

There are some difficulties in phycocyanin extraction because of multilayered cell walls and large amounts of contaminants (Stewart and Farmer 1984). Several methods have been reported for successful purification of C-PC (Bermejo et al. 2006; Boussiba and Richmond 1979; Chen et al. 2006; Patil et al. 2006; Santiago-Santos et al. 2004; Zhang and Chen 1999), but these methods comprised multiple steps and are time consuming, which may lead to increase in production costs and limit their widespread application.

In the present study we have described a one-step anion exchange chromatography with pH gradient elution for purification of C-Phycocyanin with high purity and recovery from *S. Platensis* (CCC540). The purity of C-PC was further demonstrated by sodium dodecyl sulfte- polyacrylamide gel electrophoresis (SDS-PAGE) and analysed by HPLC.

## Materials and methods

### Growth and maintenance of culture

*Spirulina platensis* (CCC540) was procured from the culture collection of Centre for Conservation and Utilization of Blue Green Algae (CCUBGA), IARI, New Delhi, India. Culture was maintained in chemically defined Z-Medium (Zarrouk 1966) at 28 ± 2 °C under a light intensity of 52–55 µmol photon m^−2^ s^−1^ and light/dark cycles of 16:8 h.

### Extraction and estimation of C-Phycocyanin

A 500 ml of homogenized log phase (15 days old) culture was centrifuged at 4,000 rpm to obtain pellet. The pellet was suspended in 100 ml of 20 mM acetate buffer containing 50 mM sodium chloride and 0.002 M sodium azide (pH-5.10). C-Phycocyanin was extracted by repeated freezing (−20 °C) and thawing at room temperature until the blue color becomes in acetate buffer (Step I). Cell debris was removed by centrifugation at 5,000 rpm for 10 min and the extract thus obtained was termed as crude extract. Amount of C-PC was measured as described by Bennett and Bogard (1973) and purity was determined by using the formulae: Purity = A_620_/A_280_

### Purification

The crude extract was subjected to a single step precipitation using 65 % (NH_4_)_2_SO_4_ (Bio Xtra, >99 %; Sigma-Aldrich) and kept overnight at 4 °C. The pellet was recovered by centrifugation at 27,000 rpm for 15 min at 4 °C and dissolved in 10 ml of the same extraction buffer and termed as ammonium sulfate extract (ASE). Ten ml of ASE was dialyzed against the extraction buffer using dialyses membrane (Dialyses membrane-70, MWCO; 12–14 kD) procured from Hi-Media. Dialyses was performed twice against 1,000 ml extraction buffer, first at room temperature and again dialysed against 1,000 ml of extraction buffer at 4 °C overnight. The resultant extract was recovered from the dialyses membrane and filtered through 0.45 µm filter.

DEAE-Cellulose from Sisco Research Laboratory (SRL) was used for anion exchange chromatography. A column (30 × 2 cm) was prepared for purifying the phycocyanin, and equilibrated with 150 ml of acetate buffer (pH-5.10). Dialyzed filtered sample (10 ml) was placed on the column. A linear gradient of acetate buffer with pH ranging from 3.76 to 5.10 was used to developed the column and elutes were collected in 5 ml fractions. Flow rate was kept 20 ml h^−1^. Absorption spectrum was also determined by scanning the sample in the range of 300–750 nm by using Specord 200 spectrophotometer (Analytikjena, Germany).

### SDS-PAGE

A 7.5 % continuous PAGE under non-denaturing conditions was carried out to reconfirm the purity of phycocyanin. The bands were visualized by Coomassie blue staining. Molecular weight of the purified phycocyanin was determined by running Novex Sharp pre stained protein marker along with the sample.

### High performance liquid chromatography

Phycobiliprotein subunit separation by HPLC was performed using a reversed phase Discovery BIO Widepore C_5_ (Supelco, Sigma Aldrich) column (250 × 4.6 mm i.d.) packed with 5 µm porous silica particles (300 angstrom pore diameter). This column was operated at a flow rate of 1 ml min^−1^ for optimum separation efficiency. All solutions were filtered through 0.5 µm membrane filter and degassed by bubbling with helium before use. Optimization of chromatographic separations was performed using a Alliance system (Waters) with 2695 separation module with auto-sampler consisting of a Waters 2998 Photo Diode-Array detector (PDA) and Waters 2475 Multi λ fluorescence detector. Excitation and emission wavelength for fluorescence detector was set at 580 and 640 nm. The Discovery BIO Widepore C_5_ column was pre-equilibrated with 20 % (v/v) aqueous acetonitrile (ACN) solution containing 0.1 % (v/v) trifluoroacetic acid (TFA). Twenty µl sample (200 µg ml^−1^) was injected and elution was performed using a linear gradient from 20 to 100 % (v/v) aqueous ACN (containing 0.1 % TFA) in 45 min. Both PDA and fluorescence detector were connected in series for the detection of biliprotein subunits.

## Results and discussion

**Fig. 1:**
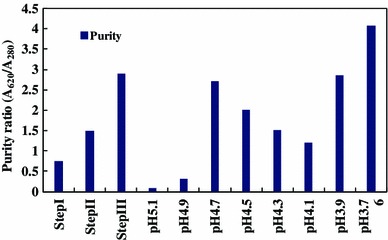
Purity of phycocyanin attained at different steps with a linear gradient of pH from 3.76 to 5.1

**Fig. 2:**
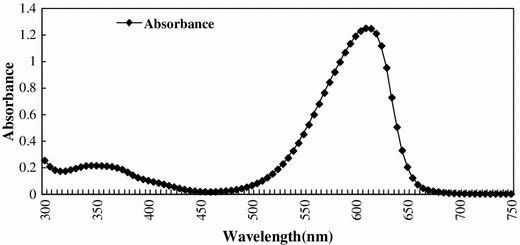
Absorbtion spectrum of purified Phycocyanin

**Fig. 3:**
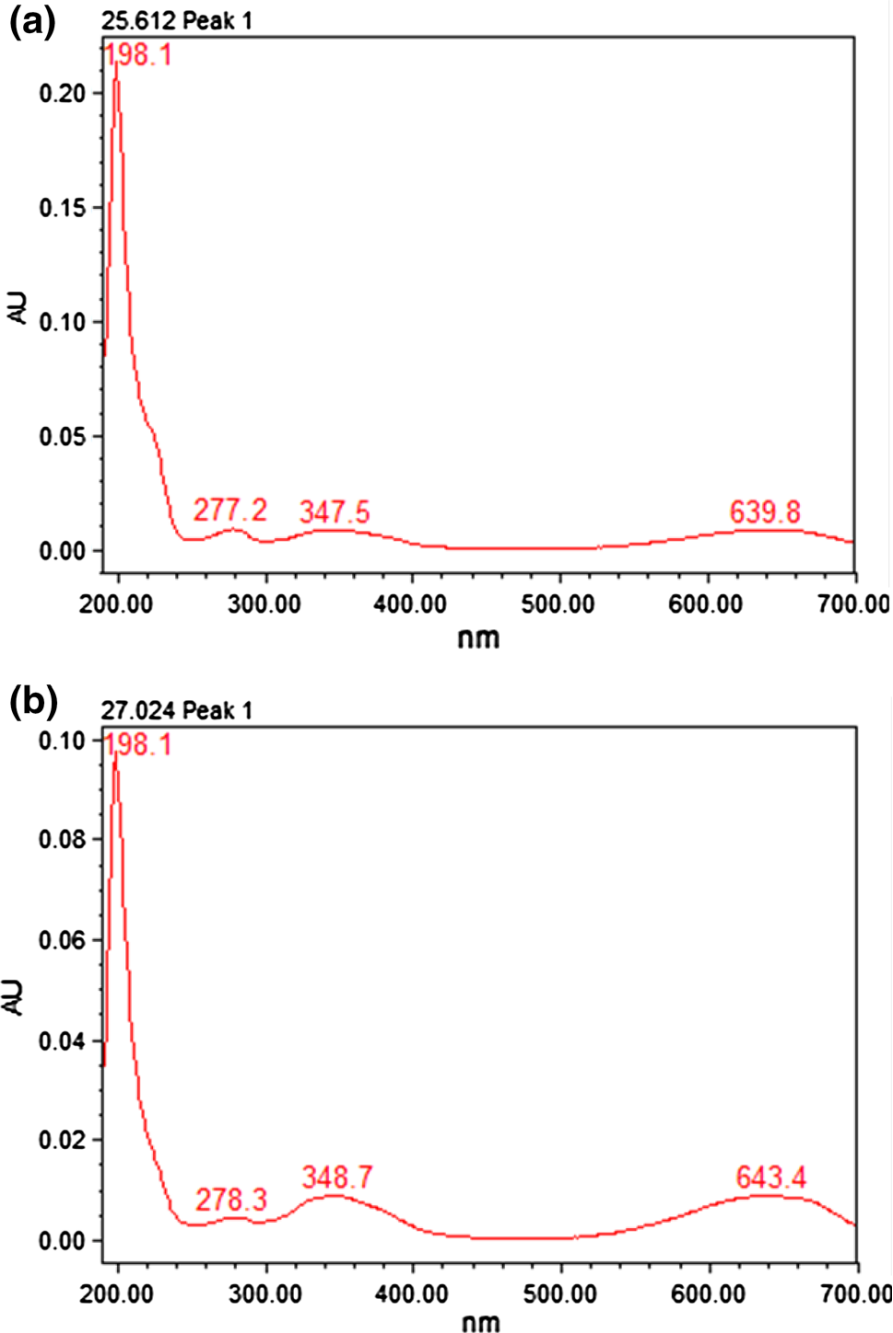
Revere phase-HPLC profile of phycocyanin from *Spirulina* strain using PDA Detector (620 nm) showing α and β subunit peaks

**Fig. 4:**
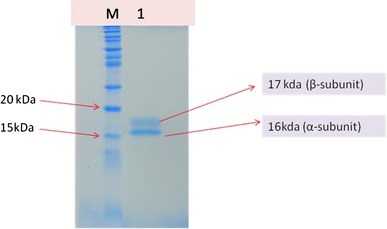
**a** Absorbtion spectra of α-subunit with RT 25.612. **b** Absorpsion spectra of β-subunit with RT 27.024

**Fig. 5:**
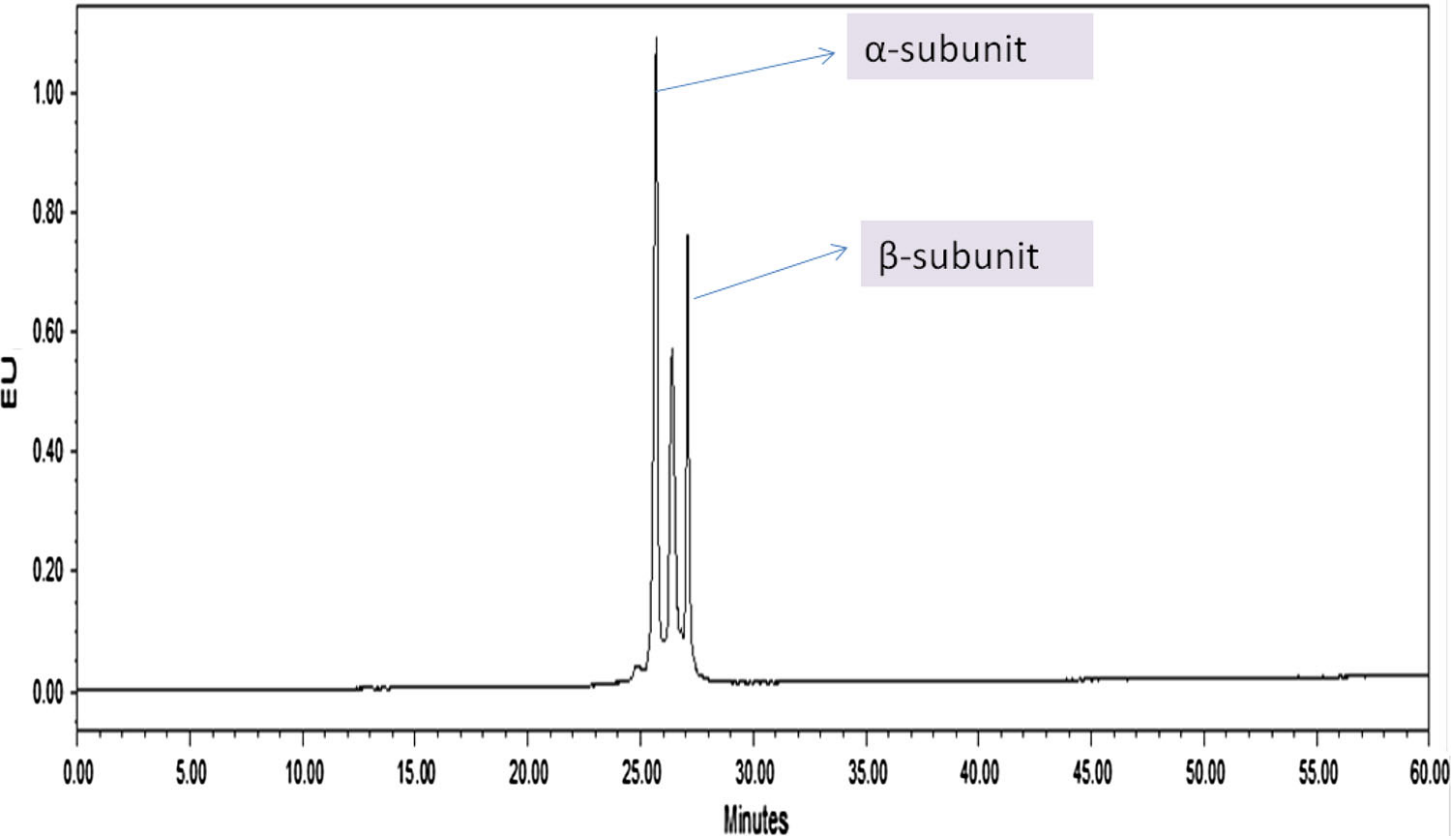
SDS-PAGE of purified phycocyanin (*M* molecular marker, *lane* 1 PC)

*Spirulina* is used as a high quality protein mainly for phycocyanin (Eriksen 2008), which is a important cyanobacterial accessory pigment having a number of industrial applications. Although a number of reports are available for the extraction and purification of phycocyanin from cyanobacterial strains (Schoenleber et al. 1984; Swanson et al. 1991). Extraction and purification of phycocyanin, was completed in four major steps: crude extract preparation (Step I), ammonium sulfate precipitation (Step II), dialyses (Step III) and anion exchange chromatography (Step IV) (Fig. 1). After ammonium sulphate precipitation the purity of phycocyanin was 1.5 and after dialyses purity were 2.93. But the final purity (A_620_/A_280_) after anion exchange chromatography was 4.58. So from crude extract purification to anion exchange chromatography, purity increased nearly six times. During the chromatographic separation, PC with maximum purity was eluted as a bright blue colored solution at pH 3.76 (Table 1). The absorption spectra of the purified PC showed a prominent peak at 620 nm (Fig. 2). Purity was also reconfirmed by the presence of single bands of α-subunit (16 kDa) and β-subunit (17 kDa) during native gel electrophoresis (Fig 5). In order to further characterize the PC purified from BGA, reverse phase HPLC was performed using C_5_ column (Fig. 3). PDA detector set at 620 and 226 nm revealed two major peaks at 25.612 and 27.024 min. When absorption spectra of these two chromatogram peaks were critically analyzed, it was found that A_620_:A_280_ for the first peak RT = 25.612 min (Fig. 4a) was approximately 1, which is due to the presence of one phycocyanobilin (PCB) chromophore, thus indicating that this peak corresponds to α subunit of PC, while the A_620_:A_280_ for the second peak RT = 27.024 min (Fig. 4b) was approximately 2, which is due to the presence of two PCB chromophores and therefore this peak corresponds to β subunit of PC.

**Table 1:** Purity and recovery ratio of phycocyanin at each purification steps

Step	Volume (ml)	PC (µg ml^−1^)	Purity (A_620_/A_280_)	Recovery (%)
Crude extract	200	77.4	0.75	100
Ammonium sulphate pptn	10	123.8	1.5	80
Dialysis	10	601.4	2.93	39
DEAE column chromatography	5	413	4.58	14

## Electronic supplementary material

Below is the link to the electronic supplementary material.

Supplementary material 1 (XLS 20 kb)

Supplementary material 2 (XLSX 13 kb)
